# Digital Eye Strain Caused by Online Education Among Children in Qassim Region, Saudi Arabia: A Cross-Sectional Study

**DOI:** 10.7759/cureus.23813

**Published:** 2022-04-04

**Authors:** Abdulrhman Aldukhayel, Samah M Baqar, Fatimah K Almeathem, Fatimah S Alsultan, Ghadah A AlHarbi

**Affiliations:** 1 Department of Family and Community Medicine, College of Medicine, Qassim University, Buraydah, SAU

**Keywords:** computer, smart devices, children, online classes, digital eye strain

## Abstract

Introduction

Online learning is the educational format adopted by schools and universities during the coronavirus disease 2019 (COVID-19) pandemic. It comprises Internet-based learning, computer-based learning, virtual classrooms, and digital collaborations. This study aims to assess digital eye strain (DES) among children attending online classes in the Qassim region, Saudi Arabia.

Materials and methods

This is a cross-sectional study conducted among children aged 3 to 18 years old in the Qassim region, Saudi Arabia. We sent out a self-administered questionnaire to parents of the targeted children by using social media, such as WhatsApp (Meta Platforms, Inc., Menlo Park, California, United States), Telegram (Telegram FZ LLC, Dubai), and Twitter (Twitter, Inc., San Francisco, California, United States). The questionnaire included questions on socio-demographic profile, smart devices being used, frequency of devices used per day before and during the lockdown, and DES.

Results

A total of 547 children were involved (50.3% males vs. 49.7% females). During online classes, the most commonly used device was tablets (51.2%). A significant increase was noticed in the use of devices during the COVID-19-related lockdown among children (p<0.001). The prevalence of DES-positive symptoms was 69.8%. The presence of DES symptoms was associated with age group (p=0.003), school level (p=0.040), device preferred for online classes (p=0.001), number of hours spent attending online classes (p=0.010), and number of hours spent using an electronic device during the lockdown (p<0.001).

Conclusion

Our study underlines a higher prevalence rate of DES among children during this time of the COVID-19 pandemic. Children who used a digital device for more than five hours during the lockdown had a greater risk of having DES than the rest.

## Introduction

Online learning was the educational format adopted by schools and universities during the coronavirus disease 2019 (COVID-19) pandemic: Internet-based learning, computer-based learning, virtual classrooms, and digital collaborations. Therefore, our children are growing up in an increasingly visually demanding world, and digital devices use has become a necessity to learn and communicate [1]. Children who spend prolonged periods in front of these devices can have eye discomfort and vision problems [2]. Digital eye strain (DES) or computer vision syndrome is an ocular problem related to the prolonged use of digital devices such as computers, smartphones, or tablets. DES is characterized by various ocular and vision-related problems [1,3].

The most common symptoms associated with DES include eye dryness, eye pain, blurred vision, headache, and neck or shoulder pain [2]. Numerous studies reported a high prevalence of DES (50-90%) among different age groups [4-6]. A cross-sectional study done among 217 Indian children attending online classes during the COVID-19 pandemic showed a high prevalence of DES (50.23%) prominently associated with the male gender and duration >5 hours in digital device use. Other risk factors include age >14 years, preferring smartphones over other devices, and restricted outdoor activities. Itching and headache were the most common symptoms [2]. Another study reported a higher prevalence of DES (92.8%) due to online classes and increased screen exposure. The most common symptoms in the study were: heaviness of eyelids and redness of the eyes [7].

Risks are associated with the duration of the online courses. A study reported that abnormal binocular vergence and accommodation parameters were more characteristic of online classes longer than four hours than online classes shorter than four hours [8]. In this regard, an Indian study concluded that online learning and smartphone use for classes longer than four hours might lead to acute acquired comitant esotropia [9]. Management commonly includes non-pharmacological actions like adjusting ergonomic practices, maintaining normal blinking, the use of appropriate lighting, careful setting of the digital device, adjusting image parameters (resolution, text size, contrast, luminance), and taking time off. Or it can be pharmacologically managed using artificial tears [3].

In our study, we aimed to assess DES among children attending online classes in the Qassim region of Saudi Arabia.

## Materials and methods

Study design, area, population, and sampling 

This is a cross-sectional study conducted among parents in Qassim, Saudi Arabia. After we obtained approval from the Committee of Research Ethics, Deanship of Scientific Research, Qassim University, Buraydah, Saudi Arabia (10-07-21), we conducted an online survey starting from January 26, 2022, to February 28, 2022. Parents participated conveniently and their consent was taken preceding the questionnaire.

Tools, size, and selection of sample 

Based on scientific literature, the estimated prevalence of DES during the COVID-19 pandemic is 45% [2,10]. Population size (for finite population correction factor (fpc)) (N): 50,000. Hypothesized percentage frequency of outcome factor in the population (p): 45%±5. The size of confidence limits on either side of the mean was 5%. Design effect (DEFF) for cluster surveys): 1. We estimated that the minimum sample size required for this study was 378; adding an additional 10% to account for the unexpected bias and missing data; we received a total of 547 participants.

We used Google Forms (Google LLC, Menlo Park, California, United States) and shared our questionnaire link via e-mails and social media like WhatsApp (Meta Platforms, Inc., Menlo Park, California, United States), Telegram (Telegram FZ LLC, Dubai), and Twitter (Twitter, Inc., San Francisco, California, United States).. The questionnaire consisted of three parts: 1) demographic data: age, gender, and class; 2) digital device details: the device used for classes, average distance, total hours using electronic devices during the lockdown, total hours using electronic devices before lockdown, total hours watching TV, total hours playing video games, and total hours attending online classes; 3) Computer Vision Syndrome Questionnaire (CVS-Q): burning in the eyes, itching, foreign body sensation, watering/tearing, excessive blinking, redness, pain, heaviness in the eyelids, dryness, blurring of vision, double vision, difficulty focusing near, halos, sensitivity to lights, headache, and worsening of eyesight. If the total score was ≥6 points, the child was considered to be suffering from digital eye strain. DES scores were further categorized as mild (DES score = 6-12), moderate (DES score = 13-18), and severe (DES score = 19-32). 

Statistical analysis 

Descriptive statistics were summarized using numbers, percentages, mean, and standard deviation. The relationship between DES and socio-demographic characteristics was conducted using the Chi-square test. A subsequent multivariate regression model was performed to determine the significant independent factor associated with positive DES with corresponding adjusted odds ratio and 95%CI. P-value <0.05 was considered statistically significant. The data analyses were performed IBM SPSS Statistics for Windows, Version 26.0 (Released 2019; IBM Corp., Armonk, New York, United States). 

## Results

In total, 547 children were involved. Table 1 describes the socio-demographic characteristics of the children. The most common age group was 8-12 years old (60.3%), with slightly more being males (50.3%) and nearly three-quarters (74%) were primary school students. The most common device used during online classes was a tablet (51.2%), while the most commonly preferred device was a laptop (35.3%). In addition, approximately 70% of the children indicated that the average distance from the devices during online classes was 10-18 inches.

**Table 1:** Sociodemographic characteristics and the use of devices by the children (n=547) * Variable with multiple response answers.

Study variables	N (%)
Age group	
3 – 7 years	114 (20.8%)
8 – 12 years	330 (60.3%)
13 – 18 years	103 (18.8%)
Gender	
Male	275 (50.3%)
Female	272 (49.7%)
School level	
Kindergarten	31 (05.7%)
Primary school	405 (74.0%)
Intermediate school	57 (10.4%)
High school	54 (09.9%)
Device use of children during online classes *	
Computer/Desktop	36 (06.6%)
Laptop	148 (27.1%)
Smartphone	202 (36.9%)
Notepad/iPad	280 (51.2%)
Preferred device for online classes	
Computer/Desktop	127 (23.2%)
Laptop	193 (35.3%)
Smartphone	65 (11.9%)
Notepad/iPad	162 (29.6%)
Average distance of device during online classes	
10-18 inches (25-46 cm)	383 (70.0%)
18-20 inches (47-50 cm)	112 (20.5%)
21-25 inches (53-64 cm)	38 (06.9%)
>25 inches (>64 cm)	14 (02.6%)

**Figure 1:**
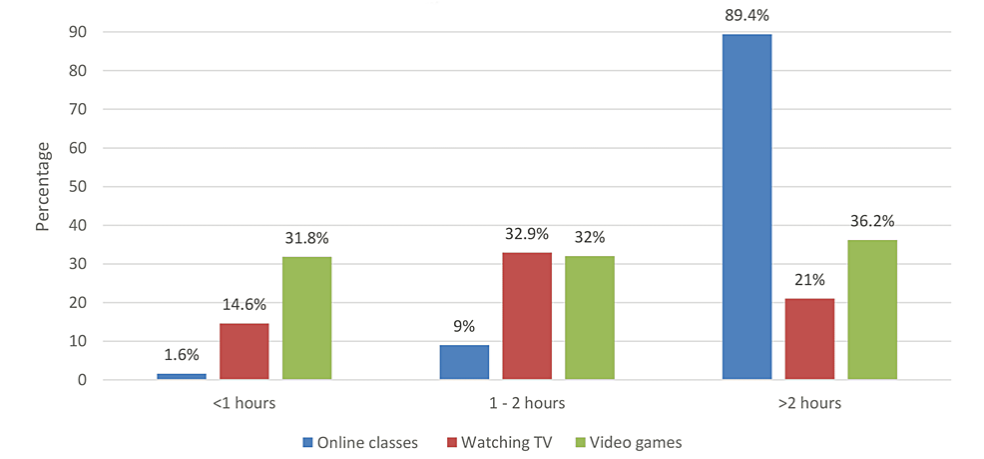
Number of hours children attended online classes, watched TV, and played video games

In Figure 1, it is seen that 89.4% of the children used a device for online classes for more than two hours, significantly higher than either watching TV (21%) or playing video games (36.2%) (p<0.001). 

**Figure 2:**
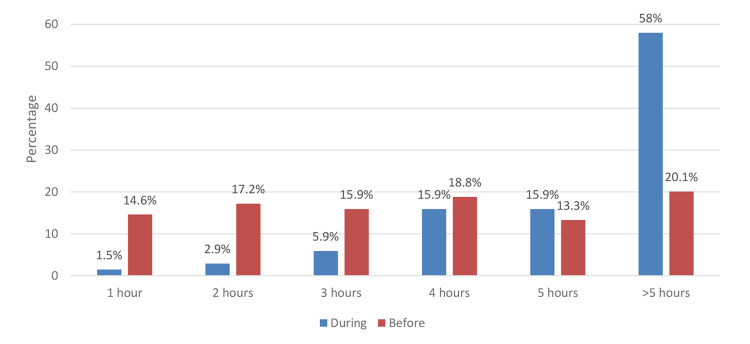
Total number of hours electronic devices were used before and during the COVID-19 lockdown COVID-19: coronavirus disease 2019

In Figure 2, during the COVID-19 lockdown, there was a higher (statistically significant) number of students using devices for more than five hours compared to pre-pandemic (p<0.001).

The descriptive statistics of the DES are represented in Table 2. It can be observed that the total mean DES score was 10.6 (SD 7.71), with 69.8% of children considered as having positive symptoms of DES while the rest (30.2%) are negative. Regarding the severity of DES, mild, moderate, and severe DES were found among 33.1%, 20.8%, and 15.9% of children showing DES symptoms, respectively.

**Table 2:** Descriptive statistics of the DES using Computer Vision Syndrome (CVS) questionnaire (n=547) DES: digital eye strain

DES parameters	N (%)
DES total score (mean ± SD)	10.6 ± 7.71
Symptoms of DES	
Positive (≥6 points)	382 (69.8%)
Negative (<6 points)	165 (30.2%)
Severity of DES	
Normal (<6 points)	165 (30.2%)
Mild (6 – 12 points)	181 (33.1%)
Moderate (13 – 18 points)	114 (20.8%)
Severe (19 – 32 points)	87 (15.9%)

We used Chi-square to measure the relationship between the symptoms of DES among the sociodemographic characteristics and the use of devices by the children (Table 3). It can be observed that the presence of DES symptoms was associated with age group (p=0.003), school level (p=0.040), device preferred for online classes (p=0.001), number of hours spent attending online classes (p=0.010), and number of hours spent using an electronic device during the lockdown (p<0.001).

**Table 3:** Relationship between the symptoms of DES among the socio-demographic characteristics and the use of devices by children (n=547) * Variable with multiple response answers. § P-value has been calculated using Chi-square test. ** Significant at p<0.05 level. DES: digital eye strain

Factor	Symptoms of DES		P-value ^§^
	Positive N (%) ^(n=382)^	Negative N (%) ^(n=165)^	
Age group			
3 – 7 years	70 (18.3%)	44 (26.7%)	0.003 **
8 – 12 years	227 (59.4%)	103 (62.4%)	
13 – 18 years	85 (22.3%)	18 (10.9%)	
Gender			
Male	181 (47.4%)	94 (57.0%)	0.040 **
Female	201 (52.6%)	71 (43.0%)	
School level			
Kindergarten	16 (04.2%)	15 (09.1%)	0.006 **
Primary school	276 (72.3%)	129 (78.2%)	
Intermediate school	45 (11.8%)	12 (07.3%)	
High school	45 (11.8%)	09 (05.5%)	
Device use of children during online classes *			
Computer/Desktop	25 (06.5%)	11 (06.7%)	0.958
Laptop	100 (26.2%)	48 (29.1%)	0.482
Smartphone	138 (36.1%)	64 (38.8%)	0.554
Notepad/iPad	200 (52.4%)	80 (48.5%)	0.406
Preferred device for online classes			
Computer/Desktop	103 (27.0%)	24 (14.5%)	0.001 **
Laptop	138 (36.1%)	55 (33.3%)	
Smartphone	35 (09.2%)	30 (18.2%)	
Notepad/iPad	106 (27.7%)	56 (33.9%)	
Average distance of device during online classes			
10-18 inches (25-46 cm)	279 (73.0%)	104 (63.0%)	0.062
>18-20 inches (47-50 cm)	71 (18.6%)	41 (24.8%)	
>20 inches (>50 cm)	32 (08.4%)	20 (12.1%)	
Number of hours attending online classes			
≤2 hours	32 (08.4%)	26 (15.8%)	0.010 **
>2 hours	350 (91.6%)	139 (84.2%)	
Number of hours watching TV			
≤2 hours	301 (78.8%)	131 (79.4%)	0.875
>2 hours	81 (21.2%)	34 (20.6%)	
Number of hours playing video games			
≤2 hours	240 (62.8%)	109 (66.1%)	0.470
>2 hours	142 (37.2%)	56 (33.9%)	
Number of hours in using the device during the lockdown			
≤5 hours	133 (34.8%)	97 (58.8%)	<0.001 **
>5 hours	249 (65.2%)	68 (41.2%)	

We subsequently performed a multivariate regression analysis to determine the significant independent predictor associated with positive DES symptoms (Table 4). Based on the result, we found that the preference of laptops for online classes and the use of devices for more than five hours were the significant independent predictors associated with positive DES symptoms. Compared to computer/desktop as device preferences, the odds of having DES were likely to decrease among children who preferred laptops by at least 60% (adjusted odds ratio (AOR)=0.416; 95% CI=0.235 - 0.737; p=0.003). On the other hand, the odds of having DES among children who used the device for more than five hours during the lockdown were 2.2 times higher compared to those who used the device for five hours or less (AOR=2.238; 95% CI=1.495 - 3.351; p<0.001). Other variables included in the model did not show a significant effect with positive DES symptoms after adjustment to the regression model, including age group, gender, school level, and the number of hours attending online classes (p>0.05). 

**Table 4:** Multivariate regression analysis to determine the independent significant factor associated with positive DES symptoms (n=547) DES: digital eye strain; AOR: adjusted odds ratio ** Significant at p<0.05 level.

Factor	AOR	95% CI	P-value
Age group			
3 – 7 years	Ref		
8 – 12 years	2.494	0.501 – 12.407	0.264
13 – 18 years	2.300	0.493 – 10.720	0.289
Gender			
Male	Ref		
Female	1.312	0.886 – 1.943	0.175
School level			
Kindergarten	Ref		
Primary school	0.923	0.133 – 6.408	0.936
Intermediate school	0.931	0.173 – 5.017	0.934
High school	1.040	0.372 – 2.906	0.941
Preferred device for online classes			
Computer/Desktop	Ref		
Laptop	0.416	0.235 – 0.737	0.003 **
Smartphone	0.771	0.480 – 1.237	0.281
Notepad/iPad	1.739	0.938 – 3.223	0.079
Number of hours attending online classes			
≤2 hours	Ref		
>2 hours	1.754	0.806 – 3.819	0.157
Total number of hours in using the devices during the lockdown			
≤5 hours	Ref		
>5 hours	2.238	1.495 – 3.351	<0.001 **

## Discussion

Due to the threat of COVID-19 infection, physical attendance in school had been halted to protect children. Mandatory online learning has emerged as an alternative for continuing education among young ones. Yet, without any appropriate guidelines, it is now the daily routine for children to spend many hours (8-12 hours per day) attending online classes using digital devices. And this leaves them vulnerable to the harmful effect caused by these gadgets without any choice [1]. 

This study attempted to evaluate the symptoms of DES suffered by children in using digital devices during online classes and determine the risk factors associated with it. Our findings revealed that the proportion of children with positive DES symptoms was 69.8%; among them, 33.1% were mild, 20.8% moderate, and 15.9% severe. These findings are almost consistent with the paper of Mohan et al. [2]. According to their reports, the prevalence of DES among children during the COVID-19 pandemic was 50.2%, with 26.3%, 12.9%, and 11.1% comprising mild, moderate, and severe DES, respectively, with consistent findings as reported by Al Tawil et al. [11], as well as Amarnath and De Ribot [12]. On the other hand, a relatively higher DES prevalence had been reported in Thailand [13]; based on their investigation, 94.8% of the lower secondary school were positive from DES. The author further emphasized that there are no available systems to regulate the use of digital devices among children and it is up to their guardians to limit the usage of digital devices. 

Studies suggest that age and use of a digital device for more than five hours per day were the independent risk factors associated with DES [2,5,7,11,12,14]. These findings are also valid in our study. Based on our results, age (8-12 years) and using a digital device for more than five hours were independently associated with DES symptoms. We also noted that the prevalence of DES symptoms was more common in females, contrary to Amarnath and De Ribot's report [12], where the odds of DES were higher on males than females. In Riyadh, Saudi Arabia [5], the use of multiple devices was the influential factor of DES, whereas in Kerala, India [15], the preferred use of a smartphone and viewing distance of digital devices (<18 inches) determined as the independent risk factors of DES. In our study, however, the preference for laptops for online classes and attending online classes for more than 2 hours were also factors that showed a significant relationship with DES. However, our regression estimates only two significant independent predictors of DES symptoms: preference for laptops and more than five hours of digital device use. The number of hours spent watching TV, playing video games, and the viewing distance are not the significant factors of DES in our study. Nevertheless, this has not been the case in a paper published in Thailand [13] in which they found viewing distance and duration of device use during the weekend to be the relevant factors of DES among children in secondary schools. 

Depending on the impact of high energy waves that can penetrate the eyes, most digital devices can harm the eyes resulting in particular ocular diseases. In our study, the most frequently used device during online classes was tablets (51.2%), while the most preferred device was a laptop. Several studies documented smartphones as the most common or preferred device during e-learning while ordinary screens (i.e., desktop) were less preferred (2.1%). In our study, the smartphone was the last choice of our children to be used in online classes. Furthermore, approximately 70% of the children in our study used devices at a distance of 10-18 inches (25-46 cm), which corroborates the studies published in India [12,15]. 

Moreover, we comprehended a significant difference in the number of hours when the child was attending online classes compared to either watching TV or playing video games. Our study showed that 89.4% of the children used digital devices for online classes for more than two hours every day, higher than the time spent playing video games (36.2%) and watching TV (21%). If collectively counted, excessive exposure to digital screens is likely to cause harm to the eyes of our children. In the Indian study [15], the majority of the children watched TV and played video games for less than one hour, which was lower than the children's viewing time in our study. When comparing the duration of exposure to digital devices between pre-COVID-19 and during COVID-19, we have learned that exposure to a digital screen for more than five hours was highly prevalent during the COVID-19 lockdown, which was consistent as discussed in the literature [2,5,12]. 

Limitations

This study was based on results from a self-administered online survey; hence, reporting bias cannot be totally eliminated. So, generalization of this study's results should be made carefully.

## Conclusions

Our study underlines a higher prevalence rate of DES among children during the COVID-19 pandemic. Children who used a digital device for more than five hours during the COVID-19 lockdown had a greater risk of having DES than the rest. A collective effort is necessary to address the high prevalence of DES among school children. Children’s awareness about the risk of prolonged use of digital devices is imperative to prevent having any symptoms associated with ocular diseases, including DES. Parents and teachers should monitor children who are becoming addicted to digital devices. An alternative plan to divert their habit is beneficial, especially during periods like the pandemic. Further research is needed to shed more light on the DES spectrum and its effect on ocular health.
